# GOLM1 Drives Colorectal Cancer Metastasis by Regulating Myeloid-derived Suppressor Cells

**DOI:** 10.7150/jca.61567

**Published:** 2021-10-21

**Authors:** Yunzhi Dang, Jiao Yu, Shuhong Zhao, Long Jin, Ximing Cao, Qing Wang

**Affiliations:** Department of Radiation Oncology, Shaanxi Provincial People's Hospital, Xi'an, 710086, China.

**Keywords:** colorectal cancer, metastasis, GOLM1, myeloid-derived suppressor cells.

## Abstract

Colorectal cancer (CRC) is the most common digestive neoplasms worldwide, metastasis and recurrence still account for the leading cause for the high mortality rate, but the exact mechanisms remain unclear. More and more evidence has indicated that the deregulation of GOLM1 plays a crucial role in cancer progression. Here, we reported a novel role of GOLM1 in promoting CRC metastasis. In this study, the expression of GOLM1 was detected in human CRC cohort. The function of GOLM1 in CRC metastasis was analyzed by *in vivo* cecum orthotopic model. We found that the expression of GOLM1 was significantly increased in CRC tissues than adjacent nontumor. Overexpression GOLM1 can promote CRC immune escape and metastasis by recruiting of myeloid-derived suppressor cells (MDSCs) at the same time. PF-04136309, a small molecule and specific inhibitor of CCR2 can largely suppressed GOLM1-mediated CRC metastasis. These results suggest that GOLM1 can promote CRC metastasis and is a prognostic biomarker in human CRC.

## Introduction

Colorectal cancer (CRC) is the third most common digestive neoplasms worldwide 1, metastasis and recurrence remains the leading cause for the high mortality rate of CRC patients. Approximately 20% of patients with CRC have metastatic disease at diagnosis 2, although great progress has been made in the treatment of CRC, the overall prognosis of is still poor for patients with metastatic CRC. Hence, it is urgently to deeply understand the underlying mechanisms of metastatic CRC.

Recent work has elucidated that inflammation plays a pivotal role in CRC initiation and metastasis 3. Human CRC often accomplished by activation of checkpoint signals, such as PD-L1/PD-1, infiltrated of immunosuppressive cells, such as tumor-associated macrophage (TAM) and myeloid-derived suppressor cells (MDSCs) 4, 5. Mounting data have shown accumulation of MDSCs, which induce local and possibly immunosuppression, is common in tumors and correlated with colorectal cancer progression 4, 6, 7. However, the ongenic signaling that drives MDSCs recruitment and activation in CRC remains poorly understood.

Golgi membrane protein 1 (GOLM1, also known as GP73 or GOLPH2), a type II transmembrane protein, is widely expressed in normal epithelial cells 8. Since Golgi apparatus mainly involved in modification and transportation of the proteins, the dysregulation of its associated proteins may lead to markedly altered cellular processes and functions 9. Accumulating evidence has indicated that the deregulation of GOLM1 gene plays critical roles in cancer progression 10, 11, metastasis 12 and immunosuppression 13. However, the function and biological function of GOLM1 in human colorectal cancer needs further research.

Here, we found that GOLM1 was overexpression in CRC and associated with poor prognosis in CRC patients. GOLM1 promoted CCL2-induced MDSCs recruitment and facilitated CRC immune escape and progression. Notably, our findings showed that CCR2 inhibitor can prevent MDSCs trafficking in CRC patients with GOLM1 overexpression. These findings reveal the mechanism of GOLM1-mediated CRC metastasis, and are helpful to develop the novel strategies for the treatment of CRC.

## Materials and Methods

### Real-time PCR

Total RNA was extracted using TRIzol Reagent (Invitrogen), and reverse transcription was performed using the Advantage RT-for-PCR Kit (Takara) according to the manufacturer's instructions. For the real-time PCR analysis, aliquots of double-stranded cDNA were amplified using a SYBR Green PCR Kit (Applied Biosystems). The cycling parameters were as follows: 95°C for 15 s, 55-60°C for 15 s, and 72°C for 15 s for 45 cycles. A melting curve analysis was then performed. The Ct was measured during the exponential amplification phase, and the amplification plots were analyzed using SDS 1.9.1 software (Applied Biosystems). For clinical tissue samples, the fold change of the target gene was determined by the following equation: 2^-ΔΔCt^ (ΔΔCt = ΔCt^tumor^ - ΔCt^nontumor^). This value was normalized to the average fold change in the normal liver tissues, which was defined as 1.0. All reactions were performed in duplicate. The primer sequences for GOLM1 sense 5'- CCGGAGCCTCGAA, AAGAGATT-3', GOLM1 antisense 5'- ATGATCCGTGTCTGGAGGTC -3'.

### Immunohistochemistry

This study was approved by the ethics the Committee of Shaanxi Provincial People's Hospital, and informed consent was written and based on the ethical guidelines of the 1975 Declaration of Helsinki. In addition, the privacy rights of human subjects were always observed. CRC specimens and matched adjacent tissues were used to construct a tissue microarray (Shanghai Biochip Co, Ltd. Shanghai, China). The tissue microarray was stained for GOLM1 (Abcam, ab109628), CD11b (Abcam, ab133357) expression. Immunohistochemistry was performed on 4-μm-thick, routinely processed paraffin-embedded sections. Briefly, after baking on a panel at 60 °C for one hour, the tissue sections were deparaffinized with xylene and rehydrated through gradient ethanol immersion. Endogenous peroxidase activity was quenched by 3% (vol/vol) hydrogen peroxide in methanol for 12 min, followed by three 3-min washes with phosphate-buffered saline (PBS). Then the slides were immersed in 0.01mol/L citrate buffer solution (pH 6.0) and placed in a microwave oven for 30 min. After washing in PBS (pH 7.4, 0.01 mol/L), the sections were incubated in a moist chamber at 4 °C overnight with the primary antibody diluted in PBS containing 1% (wt/vol) bovine serum albumin. Negative controls were performed by replacing the primary antibody with preimmunize mouse serum. After three 5 min washes with PBS, the sections were treated with a peroxidase-conjugated second antibody (Santa Cruz) for 30 min at room temperature, followed by additional three 5 min washes with PBS. Reaction product was visualized with diaminobenzidine for 2 min. Images were obtained under a light microscope (Olympus, Japan) equipped with a DP70 digital camera.

Analyses were performed by two independent observers who were blinded to the clinical outcome. The immunostaining intensity was scored on a scale of 0 to 3:0 (negative), 1 (weak), 2 (medium) or 3 (strong). The percentage of positive cells was evaluated on a scale of 0 to 4:0 (negative), 1 (1%-25%), 2 (26%-50%), 3 (51%-75%), or 4 (76%-100%). The final immuno-activity scores were calculated by multiplying the above two scores, resulting an overall score which range from 0~12. Each case was ultimately considered “negative” if the final score ranges from 0~3, and “positive” if the final score ranges from 4~12.

### Western Blotting

For Western blotting assay, the lysed cells protein was separated on SDS-PAGE and transferred onto polyvinylidene difluoride membrane. The nonspecific binding was blocked with 10% non-fat milk for one hour. The membranes were incubated with specific antibody overnight at 4°C. Western blotting of β-actin on the same membrane was used as a loading control. Antibody against GOLM1 (Abcam, ab109628) was purchased from Santa Cruz. Antibody against β-actin (A1978) was purchased from sigma. The membranes were then washed with PBS 3 times and incubated with an HRP-conjugated secondary antibody. Proteins were visualized using a Immobilon^TM^ Western Chemiluminescent HRP substrate (Millipore, USA).

### Animal experiment

All animal studies were approved by the Committee on the Use of Live Animals in Teaching and Research, Shaanxi Provincial People's Hospital. Five-weeks-old C57BL/6 male mice were raised in specific pathogen-free conditions in accord with the institutional guidelines for animal care. A metastatic colorectal model in mice was established according to the existing protocol. In brief, mouse MC38 cells (4.0×10^6^) in the 100 μl of phosphate-buffered saline were subcutaneously injected into the cecal wall of C57BL/6 mice under anesthesia (10 for each group). The *in vivo* tumor formation and metastases were monitored using the bioluminescence. For *in vivo* signal detection, D-luciferin (Perkin-Elmer) at 100 mg/kg was injected intraperitonially into the nude mice. Bioluminescent images were captured using an IVIS 100 Imaging System (Xenogeny). At the 9 weeks, the mice were sacrificed and the livers and lungs were collected and underwent histological examination. Each experiment was repeated three times and we showed the representative data of three independent experiments.

### Flow Cytometric Analysis

Cells were incubated with anti-mouse CD16/CD32 purified antibody (#101302, clone 93, Biolegend) at room temperature to block nonspecific antibodies. Then 1×10^6^ cells were stained with fluorophore-conjugated antibodies for 30 minutes at room temperature, matched isotype antibodies were used as control. Data were analyzed by Flowjio_V10 software (TreeStar, Ashland, OR). Antibodies against CD11b (FITC, #101205), CD45 (PE/Cy7, #103113), Ly-6G/Ly-6C(Gr-1) (PE, #108407) were purchased from biolegend.

### Statistical analysis

Statistics were calculated with SPSS software (version 20.0). P values were statistically analyzed by the χ^2^ test for categorical variables and by Student's test for quantitative data. The recurrence and survival data were analyzed by the Kaplan-Meier method and log-rank test. Cox proportional hazards model was used for univariate and multivariate analyses. Differences were considered statistically significant when p < 0.05.

## Results

### Elevated expression of GOLM1 in colorectal cancer positively correlates with poor prognosis

**Figure 1:**
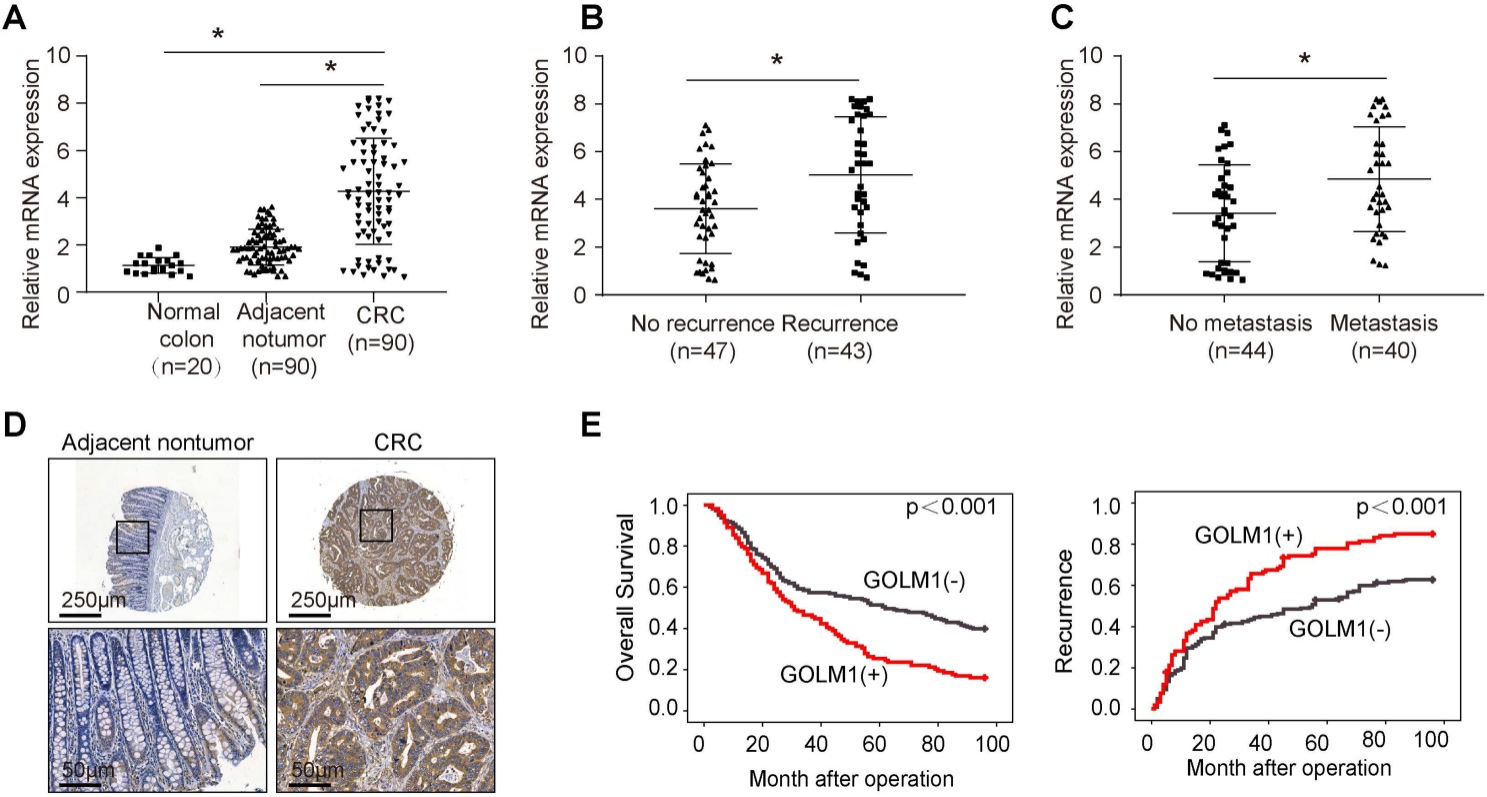
** Elevated expression of GOLM1 correlated with poor prognosis in CRC.** (A-C) Real time PCR analysis GOLM1 mRNA expression in normal colon tissues(n=20) and paired adjacent nontumor and CRC (n=90), in CRC tissues with (n=43) or without (n=47) recurrence, and in CRC tissues with (n=40) or without (n=44) metastasis. (D) Representative immunohistochemical staining of GOLM1 expression. (E) The association between GOLM1 expression with overall survival and recurrence in CRC cohort.

To determine the clinical relevance of GOLM1 expression in CRC, we examined the expression of GOLM1 by RT-PCR in a cohort of 90 paired CRC and adjacent nontumor specimens, and 20 normal colorectal epithelial specimens. We found that the mRNA levels were significantly increased in CRC compared with adjacent nontmor tissues and normal colon tissues (Figure 1A). Furthermore, it is demostrated that GOLM1 protein levels with recurrence or mtatstasis CRC were much higher than patients without recurrence or metastasis (Figure 1B-C). These data showed a close association of GOLM1 with CRC metastasis. To further determine prognostic value of GOLM1 in CRC, we analyzed the GOLM1 protein level in a tissue microrray by IHC study in 341 CRC tissues with complete clinicopathological features and complete follow-up data. We found that GOLM1 is expressed both in adjacent nontumor tissues and CRC species. However, the statistical analysis showed GOLM1 is highly expressed in CRC species than adjacent nontumor tissues (Figure 1D and Supplementary Figure S1). We found that the elevated GOLM1 expression was positively associated with tumor invasion, lymph node metastasis and AJCC stage (Table 1).

Survival analysis revealed that patients with elevated GOLM1 expression showed poorer overall survival and higher recurrence rate than patients with low GOLM1 expression (Figure 1E). In Cox proportional hazards regression analyses, GOLM1 expression was an independent predictor for patient's high recurrence rate and poor overall survival (Table 2). Based on the above findings, these indicate that the elevated overexpression is significantly associated with poor prognosis of CRC.

### Overexpression of GOLM1 promotes colorectal cancer progression

**Figure 2:**
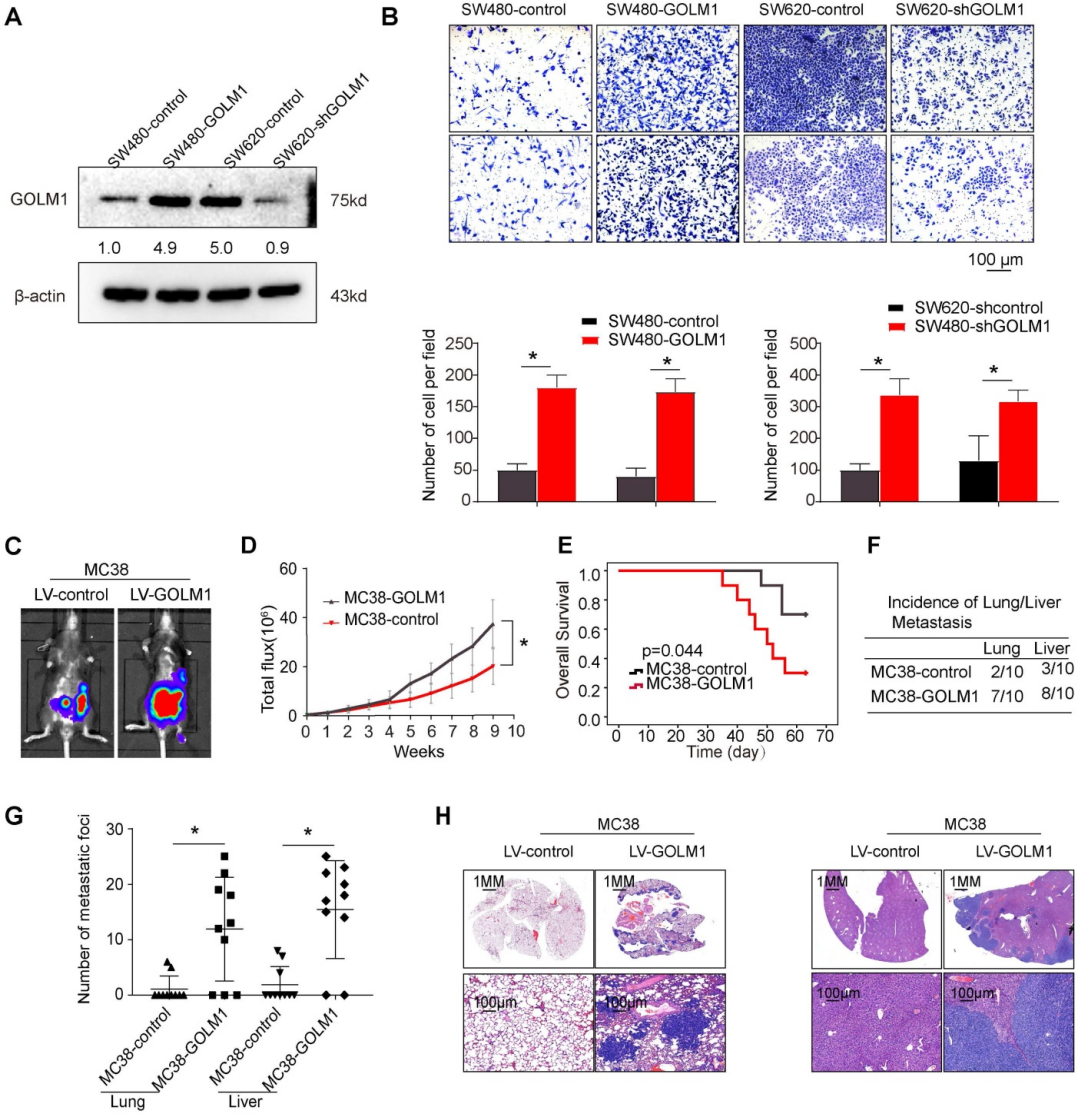
** GOLM1 promotes CRC invasion and metastasis *in vivo* and *in vitro*.** (A) GOLM1 expression in SW480 and SW620 cells by Western blot. (B) Transwell assays indicated that upregulation of GOLM1 promotes CRC migration and invasion, and depletion of GOLM1 inhibits the migration and invasion potentials of CRC cells. (C-H) Metastasis assays in the immune-competent C57BL/6 mice. Bioluminescent imaging(C), Bioluminescent signals(D), Overall survival(E), The incidence and number of lung and liver colonization(F-G), and HE (H). * P<0.05.

The above data suggest that GOLM1 may play a key role in CRC metastasis. Accordingly, we evaluated the functions of GOLM1 on cell migration in a gain of function experiments both *in vitro* and *in vivo* assays. Transwell assay showed that overexpression of GOLM1 can promote CRC invasion and migration, while downregulation of GOLM1 can suppress the invasion and migration abilities of CRC cells (Figure 2A-B). To further investigate the impact of GOLM1 on the metastasis, we established the cecal metastasis model using MC38-GOLM1 cells and its corresponding control cells. *In vivo* orthotopic implantation of immunocompetent C57BL/6 mice assay showed that GOLM1 upregulation increased the bioluminescence intensity, lung metastasis incidence, while shorten the overall survival of the MC38 group (Figure 2C-H). These above results indicate GOLM1 can promote CRC metastasis both *in vitro* and* in vivo*.

### GOLM1 promotes CRC progression by recruitment of MDSCs

**Figure 3:**
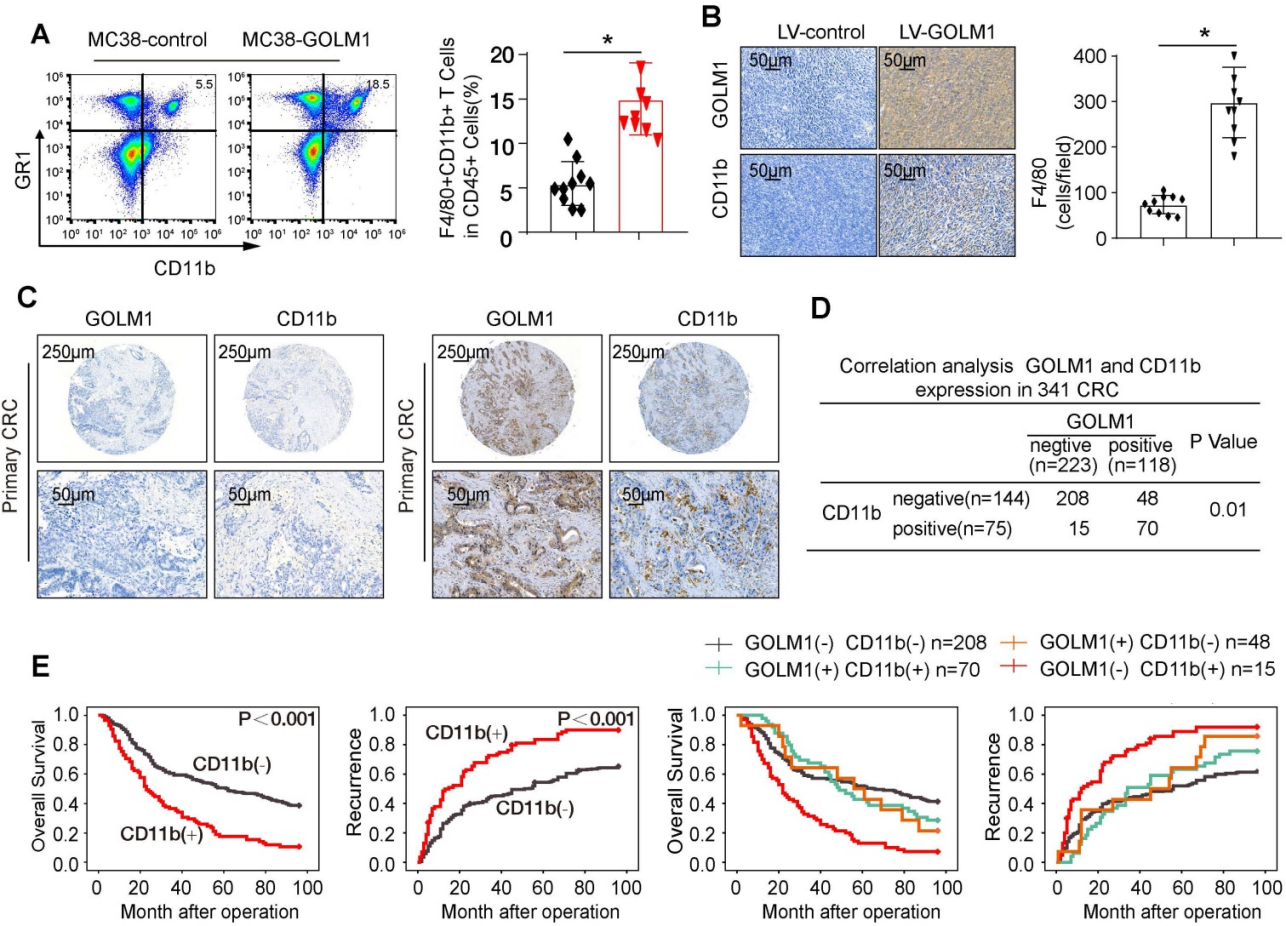
** GOLM1 promotes CRC progression in immunocompetent mice by recruitment of MDSCs.** (A) The infiltration of CD11b+GR1+ MDSCs were determined by flow cytometry. (B) The MDSCs infiltration of the indicated groups was evaluated by IHC staining. (C) IHC staining analysis of GOLM1 and CD11b expression in CRC cohort. (D) The expression relationship between GOLM1 and CD11b in CRC cohort. (E) Kaplan-Meier analysis of the association between the expression of CD11b or GOLM1 /CD11b coexpression and recurrence or overall survival in CRC cohort.

Mounting data have shown accumulation of MDSCs, which induce local and possibly immunosuppression, is correlated with CRC progression 7. We examined the infiltration of MDSCs by flowcytometric analysis and IHC staining. The results showed that the number of MDSCs markedly elevated in MC38-GOLM1 tumor than MC38-control cells (Figure 3A-B). To verify clinical relevance, we further investigated the expression of CD11b in CRC cohort. The representative images of CD11b were showed in Figure 3C. Correlation assay suggested that GOLM1 positively correlated with CD11b (Figure 3D).

Survival analysis indicated that CRC patients with CD11b expression showed poorer overall survival and elevated recurrence rates than patients without CD11b expression. Furthermore, CRC patients that showed positive coexpression of GOLM1/CD11b had the lowest survival rate and the highest recurrence rate (Figure 3E).

### The CCR2 inhibitor PF-04136309 can inhibit GOLM1-mediated CRC metastasis

**Figure 4:**
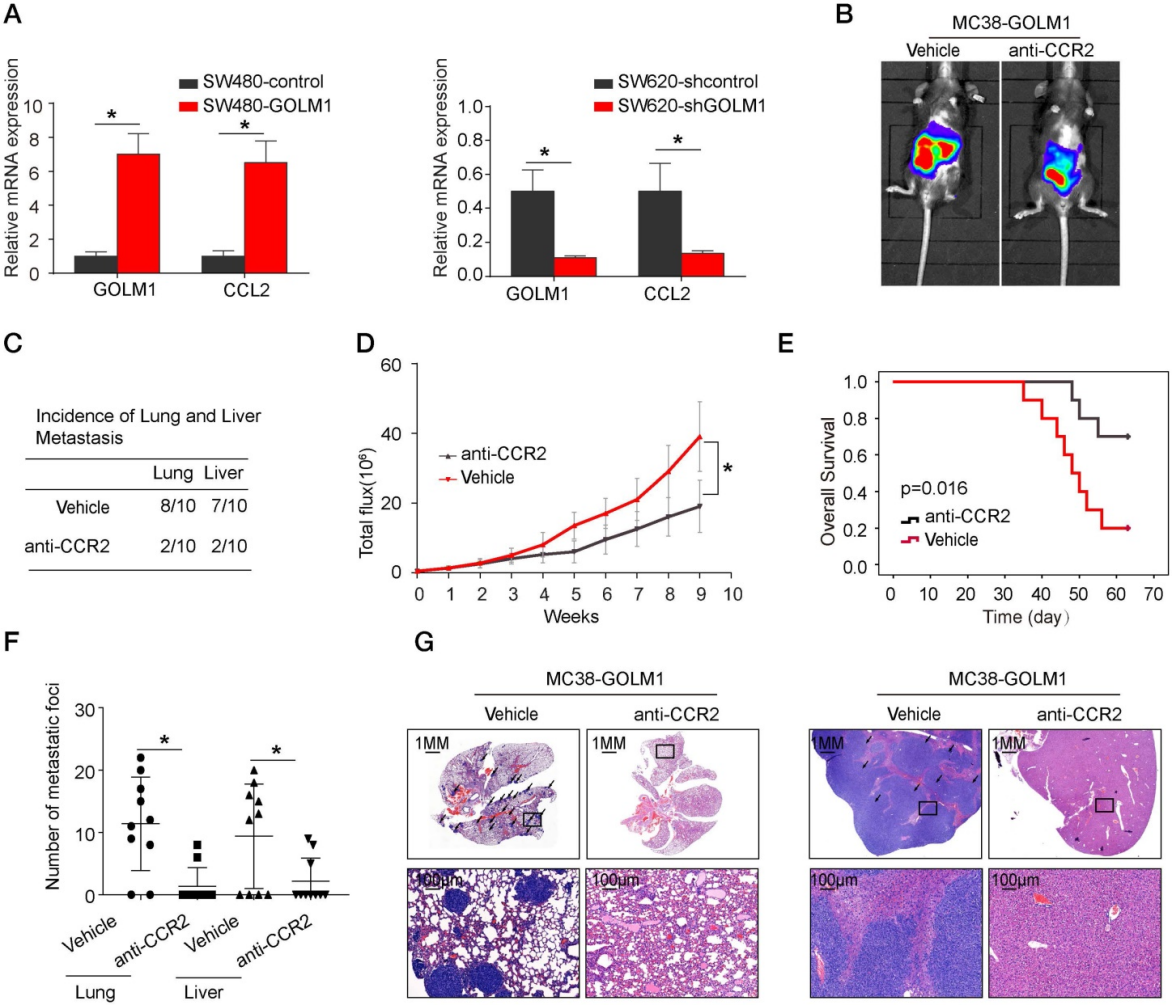
** The CCR2 inhibitor PF-04136309 can inhibit GOLM1-mediated CRC metastasis.** (A) GOLM1 and CCL2 expression in the indicated cell by Real-time PCR analysis. (B-G) PF-04136309 (100 mg/kg, subcutaneously, twice, daily, for 9 weeks) reduced the MC38-GOLM1 cells tumor metastasis. Bioluminescence images(B), The incidence of lung and liver colonization (C), Bioluminescence signals(D), Overall survival (E), The number of lung and liver colonization(F), HE staining (G). * P<0.05.

To study the underlying mechanism of CRC recruit MDSCs, we selected the CRC cell lines with low expression of GOLM1 and constructed SW480-GOLM1 stable cell lines after transfection with lentivirus. By PCR microarray of chemokines and their receptors, we found that GOLM1 overexpression promoted transcriptome changes of a series of chemokines and their receptors such as CCL2, CXCL5, CXCL12, CCL4 (Table 3). Considering the important role of CCL2 in cancer progression, we focused on CCL2 for further study. Real time assay showed that overexpression of GOLM1 markedly upregulated CCL2 expression, whereas downregulation of GOLM1 can reduce the expression levels of CCL2 (Figure 4A).

Previous study showed that CCL2 recruits immune suppressor cells MDSCs and TAMs to tumor sites by binding to CCR2 receptor. Then, we investigate whether inhibition the CCL2/CCR2 signal can suppress CRC progression. PF-04136309 14, an antagonist of CCR2, can specifically inhibit the binding of the ligand CCL2 to its receptor CCR2, which may lead to the inhibition of CCR2 activation and signal transduction. We aimed to study whether PF-04136309 treatment affects GOLM1-CCL2 signaling-medicated CRC metastasis. *In vivo* study showed that PF-04136309 can significantly reduce lung and liver metastasis, metastasis, while prolonged the survival of MC38-GOLM1 group compared to that of the control group.

## Discussion

Colorectal cancer is one of the most common digestive system neoplasms with high mortality rate 15. Although great improvements had been made in screening and treatments, the prognosis is still poor for metastatic CRC. Metastasis still accounts for the main cause of poor clinical outcome of CRC patients 16. Hence, a deeply study of the underlying mechanisms of metastasis in CRC is crucial to find new treatment strategies for colorectal cancer. Deregulated GOLM1 functioned as an oncogene and correlated with poor prognosis in many types of cancers, such as hepatocellular carcinoma 12, 17, prostate cancer 18, gastric cancer 11, esophagus cancer 19 and so on. In this study, we found that GOLM1 was overexpression in CRC and the elevated GOLM1 correlated with a higher recurrence rate, shorter overall survival and more aggressive tumor phenotype. GOLM1 upregulation increased the bioluminescence intensity, lung metastasis incidence, while shorten the overall survival of the MC38 group *in vivo*. However, the underlying mechanism of GOLM1 functions as an oncogene in CRC is still unclear. In our previous work, we indeed have studied whether GOLM1 can promote CRC metastasis in both immunocompetent C57BL/6 mice and immune-deficiency nude mice. The SW480-GOLM1 and indicated control cell was injected into the cecal wall of nude mice, while MC38-GOLM1 and indicated control cell was injected into the cecal wall of C57BL/6 mice. These results showed both in nude mice and in C57BL/6 mice, upregulation of GOLM1 can promote CRC metastasis (Figure 2 and Supplementary Figure 2). However, compared with nude mice, GOLM1 promoted CRC metastasis more significantly in C57BL/6 mice. These results indicated that an intact immune system is essential for GOLM1 promote CRC metastasis. However, the underlying mechanism needs further study.

Immune invasion is one of the major hallmarks of cancers 20, often accomplished by expansion of PD-1/PD-L1 axis or the infiltration of immunosuppressive cells such as MDSCs 21-24. MDSCs represent the universal immunosuppressive population in many pathologic conditions including cancer 24. Accumulating evidence in recent years has even highlighted the expansion of MDSCs in human CRC as a major barrier to antitumor immunity and escape immune surveillance 24, 25. More importantly, MDSCs can in turn suppress CD8+ T cell cytotoxicity. Our *in vivo* study showed that GOLM1 promote CRC metastasis by recruitment of MDSCs. In clinical sample, correlation assay suggested that GOLM1 positively correlated with CD11b. Furthermore, CRC patients with positive coexpression of GOLM1/CD11b had the lowest survival rate and the highest recurrence rate.

Our results showed that GOLM1 can upregulate the expression of CCL2, which leads to the recruitment of MDSCs. CCL2, highly expressed in many types of cancers, can recruit monocyte and macrophages into tumors to promote tumor cell survival and contribute immune evasion. Previous studies reported that PF-04136309, a specific CCR2 inhibitor, can augment antitumor immunity and improved response to chemotherapy 26. In this study, we found that PF-04136309 can significantly inhibited MDSCs cells recruitment. In addition, *in vivo* PF-04136309 can reduce the metastasis rate of CRC, while prolonged the survival time of C57 mice.

In conclusion, we found that CRC was overexpression in CRC and associated with poor prognosis. GOLM1 promoted induced MDSCs recruitment by CCL2/CCR2 pathway to facility CRC immune escape and progression. PF-04136309, a small molecule and specific inhibitor of CCR2, inhibit GOLM1-induced CRC metastasis and MDSCs recruitment.

## Supplementary Material

Supplementary figures.

## Figures and Tables

**Table 1:** Correlation between GOLM1 expression and clinicopathological characteristics of CRC in cohort of human CRC tissues

Clinicopathological variables		Tumor GOLM1 expression		*P* Value
		Negative (n=223)	Positive (n=118)	
Age	≤60	69	29	0.161
	>60	154	89	
Gender	female	91	61	0.054
	male	132	57	
Tumor size	≤5cm	91	38	0.119
	>5cm	132	80	
Tumor differentiation	Well or moderate	138	65	0.224
	poor	85	53	
Tumor invasion	T1-T3	190	88	0.016
	T4	33	30	
Lymph node metastasis	Absent	147	63	0.024
	Present	76	55	
Distant metastasis	Absent	194	94	0.075
	Present	29	24	
AJCC stage	I-II	143	60	0.017
	III-IV	80	58	

**Table 2:** Univariate and multivariate analysis of factors associated with survival and recurrence in two independent cohorts of human CRC.

Clinical Variables	Time to Recurrence		Overall Survival	
	HR (95% CI)	P value	HR (95% CI)	P value
**Univariate analysis**				
Age (≤60 vs > 60)	1.027 (0.771-1.368)	0.853	1.146 (0.877-1.496)	0.766
Gender (female vs male)	0.934 (0.724-1.206)	0.601	0.927 (0.716-1.199)	0.562
Tumor size (≤5 cm vs >5 cm)	1.073 (0.826-1.395)	0.596	0.597 (0.439-0.821)	0.318
Tumor differentiation (well/moderate vs poor)	0.50 (0.387-0.647)	<0.001	0.474 (0.366-0.614)	<0.001
Tumor invasion (I-II vs III)	0.585 (0.432-0.791)	0.001	0.597 (0.439-0.821)	0.001
Lymph node metastasis (absent vs present)	0.190 (0.144-0.250)	<0.001	0.174 (0.131-0.231)	<0.001
Distant metastasis (absent vs present)	0.121 (0.085-0.173)	<0.001	0.099(0.069-0.1143)	<0.001
AJCC stage (I-II vs III)	0.173 (0.131-0.229)	<0.001	0.159 (0.120-0.210)	<0.001
GOLM1 expression (negative vs positive)	0.569 (0.439-0.738)	<0.001	0.555(0.427-0.7220)	<0.001
**Multivariate analysis**				
Tumor differentiation (well/moderate vs poor)	0.844 (0.632-1.127)	0.251	0.851(0.634-1.140)	0.279
Tumor invasion (I-II vs III-IV)	0.748 (0.540-1.035)	0.080	0.789(0.567-1.098)	0.160
Lymph node metastasis (absent vs present)	1.175 (0.536-2.576)	0.686	0.937(0.428-2.055)	0.872
Distant metastasis (absent vs present)	0.334 (0.225-0.497)	<0.001	0.275(0.183-0.412)	<0.001
AJCC stage (I-II vs III)	0.334 (0.244-0.497)	<0.001	0.223 (0.099-0.505)	<0.001
GOLM1 expression (negative vs positive)	0.194 (0.086-0.438)	0.002	0.641 (0.485-0.846)	0.002

**Table 3:** Chemokines and Receptors RT^2^ Profiler PCR Array of SW480-GOLM1 vs SW480 -control

Gene	Description	SW480-GOLM1 (fold change)
CCL2	Chemokine (C-C motif) ligand 2	4.95
CXCL5	Chemokine (C-X-C motif) ligand 5	4.31
CXCL12	Chemokine (C-X-C motif) ligand 12	4.15
CCL4	Chemokine (C-C motif) ligand 4	3.14
CXCL2	Chemokine (C-X-C motif) ligand 2	3.45
CCR7	Chemokine (C-C motif) receptor 7	3.12
CCL5	Chemokine (C-C motif) ligand 5	2.85
CCL22	Chemokine (C-C motif) ligand 22	2.85
CCR9	Chemokine (C-C motif) receptor 9	2.84
ACKR1	Duffy blood group, chemokine receptor	2.74
CCR4	Chemokine (C-C motif) receptor 4	2.66
CXCR4	Chemokine (C-X-C motif) receptor 4	2.56
CCL26	Chemokine (C-C motif) ligand 26	2.55
ACKR4	Chemokine (C-C motif) receptor-like 1	2.54
CX3CR1	Chemokine (C-X3-C motif) receptor 1	2.34
IL16	Interleukin 16	2.32
CCR8	Chemokine (C-C motif) receptor 8	2.31
CXCL9	Chemokine (C-X-C motif) ligand 9	2.31
CKLF	Chemokine-like factor	2.21
CCL20	Chemokine (C-C motif) ligand 20	2.2
CXCR3	Chemokine (C-X-C motif) receptor 3	2.15
CCL19	Chemokine (C-C motif) ligand 19	2.11
CXCL10	Chemokine (C-X-C motif) ligand 10	2.1
CXCL14	Chemokine (C-X-C motif) ligand 14	1.95
ACKR3	Chemokine (C-X-C motif) receptor 7	1.85
PPBP	Pro-platelet basic protein (chemokine (C-X-C motif) ligand 7)	1.85
CCR2	Chemokine (C-C motif) receptor 2	1.83
CMTM2	CKLF-like MARVEL transmembrane domain containing 2	1.83
TLR4	Toll-like receptor 4	1.81
CCR10	Chemokine (C-C motif) receptor 10	1.75
CXCL6	Chemokine (C-X-C motif) ligand 6 (granulocyte chemotactic protein 2)	1.66
CMTM3	CKLF-like MARVEL transmembrane domain containing 3	1.63
CCL24	Chemokine (C-C motif) ligand 24	1.59
CCL25	Chemokine (C-C motif) ligand 25	1.56
CCL27	Chemokine (C-C motif) ligand 27	1.55
CCL14	Chemokine (C-C motif) ligand 14	1.53
CCR5	Chemokine (C-C motif) receptor 5	1.51
CCL11	Chemokine (C-C motif) ligand 11	1.51
CCL15	Chemokine (C-C motif) ligand 15	1.47
CCL13	Chemokine (C-C motif) ligand 13	1.47
CXCR2	Chemokine (C-X-C motif) receptor 2	1.46
TLR2	Toll-like receptor 2	1.45
CXCL13	Chemokine (C-X-C motif) ligand 13	1.44
CXCL3	Chemokine (C-X-C motif) ligand 3	1.44
CXCL1	Chemokine (C-X-C motif) ligand 1 (melanoma growth stimulating activity, alpha)	1.44
CCR3	Chemokine (C-C motif) receptor 3	1.44
CCL1	Chemokine (C-C motif) ligand 1	1.41
CXCR1	Chemokine (C-X-C motif) receptor 1	1.33
CXCL11	Chemokine (C-X-C motif) ligand 11	1.23
SLIT2	Slit homolog 2 (Drosophila)	1.23
CCRL2	Chemokine (C-C motif) receptor-like 2	1.21
CCL7	Chemokine (C-C motif) ligand 7	1.10
IL1B	Interleukin 1, beta	1.03
CXCR5	Chemokine (C-X-C motif) receptor 5	0.
CCL8	Chemokine (C-C motif) ligand 8	-1.17
CCL21	Chemokine (C-C motif) ligand 21	-1.22
TYMP	Thymidine phosphorylase	-1.23
CCL23	Chemokine (C-C motif) ligand 23	-1.33
CMKLR1	CHEMOKINE-LIKE RECEPTOR 1	-1.33
CCL3	Chemokine (C-C motif) ligand 3	-1.34
GPR17	G protein-coupled receptor 17	-1.44
PF4V1	Platelet factor 4 variant 1	-1.66
CXCL16	Chemokine (C-X-C motif) ligand 16	-1.84
ACKR2	Chemokine binding protein 2	-1.85
TNF	Tumor necrosis factor	-1.89
CCL28	Chemokine (C-C motif) ligand 28	-2.11
CCL16	Chemokine (C-C motif) ligand 16	-2.14
C5	Complement component 5	-2.32
FPR1	Formyl peptide receptor 1	-2.33
CMTM1	CKLF-like MARVEL transmembrane domain containing 1	-2.34
CCR1	Chemokine (C-C motif) receptor 1	-2.39
CCL17	Chemokine (C-C motif) ligand 17	-2.44
XCL1	Chemokine (C motif) ligand 1	-2.45
C5AR1	Complement component 5a receptor 1	-2.45
XCR1	Chemokine (C motif) receptor 1	-2.45
CCL18	Chemokine (C-C motif) ligand 18 (pulmonary and activation-regulated)	-2.85
CMTM4	CKLF-like MARVEL transmembrane domain containing 4	-2.88
IL8	Interleukin 8	-3.11
XCL2	Chemokine (C motif) ligand 2	-3.11
CCR6	Chemokine (C-C motif) receptor 6	-3.12
CXCR6	Chemokine (C-X-C motif) receptor 6	-3.15
HIF1A	Hypoxia inducible factor 1, alpha subunit (basic helix-loop-helix transcription factor)	-3.31
CX3CL1	Chemokine (C-X3-C motif) ligand 1	-3.55
IL4	Interleukin 4	-3.65

## Abbreviations

CRC: Colorectal cancer
HE: hematoxylin and eosin
IHC: immunohistochemistry
BLI: bioluminescent imaging
GOLM1: Golgi membrane protein 1
MDSCs: myeloid-derived suppressor cells
