# Supplementary material for: GOLM1 Drives Colorectal Cancer Metastasis by Regulating Myeloid-derived Suppressor Cells
Source: J Cancer. 2021 Oct 21;12(23):7158–66. doi: 10.7150/jca.61567 (PMC8558645; doi:10.7150/jca.61567)
Supplement: Supplementary file 1 — Supplementary figures. [file jcav12p7158s1.pdf]

Supplementary Materials

GOLM1 Drives Colorectal Cancer Metastasis by Regulating Myeloid-derived Suppressor Cells

Yunzhi Dang✉, Jiao Yu, Shuhong Zhao, Long Jin, Ximing Cao, Qing Wang

Department of Radiation Oncology, Shaanxi Provincial People's Hospital, Xi'an, 710086, China

Corresponding author: Dr. Yunzhi Dang

Department of Radiation Oncology, Shaanxi Provincial People's Hospital, Xi'an 710086, China

Tel: +86-29-8525 2140

Email: dangyunzhi@xiyi.edu.cn

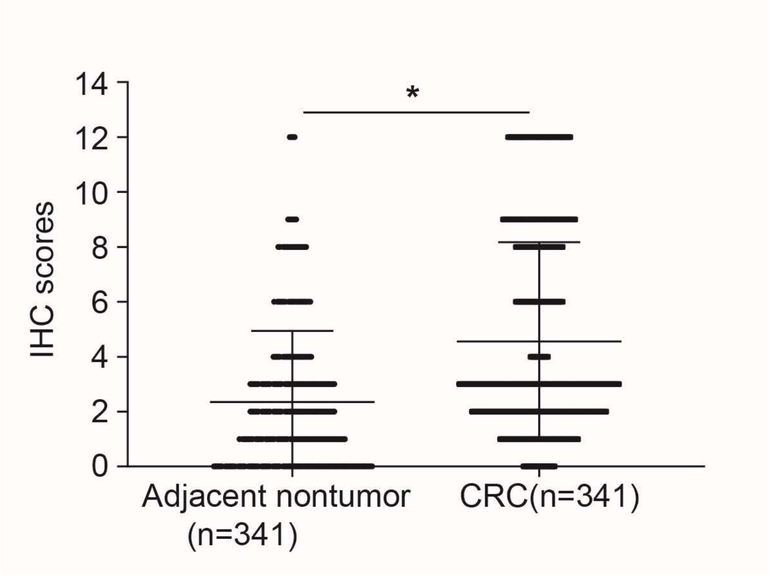

Supplementary Figure 1. IHC scores of GOLM1 in human CRC tissues and adjacent nontumor tissues.

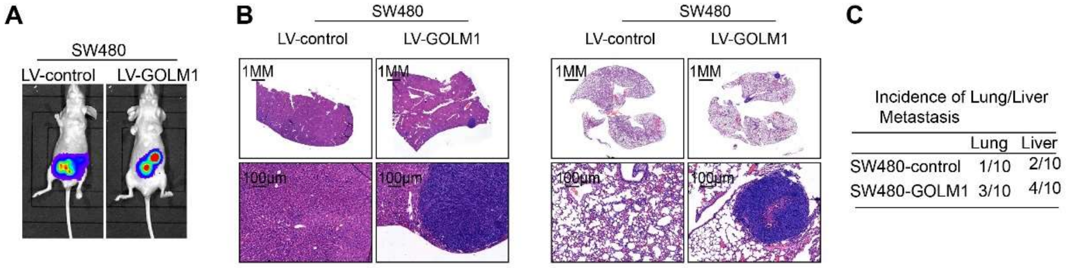

Supplementary Figure 2. GOLM1 promotes CRC metastasis in nude mice.

(A-B) Metastasis assays in the immune-deficiency nude mice. Bioluminescent imaging(A), HE (B), and the incidence and number of lung and liver metastasis (C).
